# Experimental Evaluation of LR-FHSS: A Comparison with LoRa

**DOI:** 10.3390/s25237209

**Published:** 2025-11-26

**Authors:** Roger Sanchez-Vital, Lluís Casals, Bernat Jara-Ortínez, Jana Bodvanski, Rafael Vidal, Eduard Garcia-Villegas, Carles Gomez

**Affiliations:** Department of Network Engineering, Universitat Politècnica de Catalunya, C/Esteve Terradas, 7, 08860 Castelldefels, Spain; lluis.casals@upc.edu (L.C.); jaraortinez.b7@gmail.com (B.J.-O.); jana.bodvanski@gmail.com (J.B.); rafael.vidal@upc.edu (R.V.); eduardo.garcia@upc.edu (E.G.-V.); carles.gomez@upc.edu (C.G.)

**Keywords:** LoRaWAN, LoRa, LR-FHSS, terrestrial, coverage, performance evaluation, Internet of Things, IoT, LPWAN

## Abstract

Long-Range Frequency Hopping Spread Spectrum (LR-FHSS) is the newest modulation in LoRaWAN, designed to overcome the scalability and coverage limits of conventional LoRa. This study provides a real-world evaluation of LR-FHSS performance, benchmarking it directly against LoRa. An outdoor campaign was conducted in urban and semi-urban scenarios in and near the city of Castelldefels using a complete LR-FHSS-enabled network and an end-device transmitting at LoRa and LR-FHSS data rates (DRs). Measurements were collected along four diverse paths, capturing key metrics such as Received Signal Strength Indicator (RSSI) and Packet Delivery Ratio (PDR). The results clearly underline the advantages of LR-FHSS; while LoRa at DR0 and DR5 quickly lost connectivity beyond 1.5–2 km, LR-FHSS, particularly at DR8 and DR10, kept reliable links at 3–4 km. LR-FHSS robustness was most evident in non-line-of-sight (NLoS) and long-range scenarios. These findings highlight LR-FHSS as a strong candidate for future IoT deployments, offering extended range and higher robustness in challenging environments.

## 1. Introduction

LoRaWAN is a prominent wireless, low-power, and long-range communication technology. LoRaWAN has positioned itself as a flagship Low-Power Wide Area Network (LPWAN) technology, and also as a popular technology to enable Internet of Things (IoT) use cases [1,2].

LoRaWAN supports a range of physical-layer (PHY) configuration choices, including several mandatory and optional data rates (DRs). The mandatory DRs, which are the most commonly used, are based on the Long Range (LoRa) modulation [3]. LoRa employs chirp spread spectrum (CSS) modulation with fixed channels, offering excellent range and low power consumption. However, in dense deployments, it can suffer from congestion due to limited channel capacity.

To enhance network capacity and communication robustness, the LoRaWAN specification has recently been enhanced with new PHY options, based on the Long Range-Frequency Hopping Spread Spectrum (LR-FHSS) modulation [4]. LR-FHSS uses frequency hopping across multiple narrowband channels. This approach may introduce slightly higher power consumption due to frequency hopping overhead and increased transmission redundancy [5], while adding implementation complexity. Nevertheless, it is designed to increase scalability and interference resistance, making it better suited for high-density networks, where it is expected to support longer communication links than LoRa.

LR-FHSS has attracted the interest of the research community [5,6,7,8,9,10,11,12,13,14,15,16,17,18,19,20,21]. Many studies aim to evaluate and/or improve the performance of LR-FHSS by using different techniques, such as mathematical analysis, simulation, and experimental approaches. However, the latter has been limited by challenges involved in setting up real-world LoRaWAN networks supporting LR-FHSS [5,6,7]. This phenomenon is especially critical, since mathematical analysis or simulation cannot always capture the characteristics of real wireless network scenarios.

In this paper, we provide an experimental evaluation of the performance of LR-FHSS and compare it with that of LoRa across a variety of real-life scenarios in urban and semi-urban environments. We focus on Packet Delivery Ratio (PDR) and Received Signal Strength Indicator (RSSI) as the main performance parameters. Our results show that LR-FHSS outperforms LoRa, achieving high PDR even in scenarios characterized by low RSSI values and/or where LoRa fails to provide connectivity. We also illustrate that if both average RSSI and DR information are known, PDR can be reasonably predicted.

The remainder of this paper is organized as follows: Section 2 overviews the related work. Section 3 describes the fundamentals of LoRaWAN and LR-FHSS. Section 4 provides our experimental evaluation, which includes the details of the considered setup and scenarios, the experimental results, and the discussion of our main findings. Section 5 points out open issues of LR-FHSS that have been identified in this study. Finally, Section 6 concludes the paper with the main remarks and future research directions.

## 2. Related Work

In this section, an overview of the literature on LR-FHSS performance evaluation is provided, with a particular focus on studies carried out in real deployments. Notably, only two articles that evaluate PDR of LR-FHSS in real environments have been made publicly available, as of the writing [6,7]. In contrast, there is ample literature of similar studies on LoRa, where a variety of scenarios, such as urban [22], indoor [23], maritime [24], rural [25], and space [26] are considered.

Performance evaluation of LR-FHSS has attracted increasing interest in recent years. Many studies are dedicated to packet delivery and error rate [6,9,10,11,12,13,14,15,16,17,18,19,20,21]. Other performance parameters considered comprise energy consumption [5,27], network capacity [10,11,13,16], and throughput [8,10,13]. However, most of the works that focus on packet delivery or error rate are based on analytical models [9,11,12,13,14,15,16,17,18] or simulation [10,17,18,19,20].

Only four works provide empirical results [6,7,16,21], the last three being the only ones that consider the impact of the distance between the end-device (ED) and the gateway (GW) on performance. More specifically, V.L. Tran et al. use city-wide coverage maps and aggregated values of RSSI and received packets to demonstrate the benefits of LR-FHSS for LoRaWAN in the AS923 band [21]. Nevertheless, the experimental setup and reported results remain insufficiently detailed, and PDR is not considered. For instance, the specific ED model is not disclosed, and the DRs employed, as well as the relationships among the evaluated performance parameters—RSSI, Signal-to-Noise Ratio (SNR), and distance—are not provided.

J. Bukhari et al. design and implement an LR-FHSS ED and a GW and, through a set of evaluations, conclude that the technology meets expectations in terms of both communication range and network scalability [16]. However, unlike our study, their work neither employs commercial products nor utilizes a fully operational LR-FHSS-enabled LoRaWAN network. Furthermore, in their real-world experiments, only DR9 is considered among the LR-FHSS DRs, and a CSS configuration is used that does not correspond to any of the standard LoRa DRs but is instead configured to match DR9’s bit rate. In addition, only three relatively short distances between ED and GW are evaluated, the largest being 1.54 km.

M. Mardones et al. evaluate PDR in an urban deployment, RSSI in a mobility test based on two bicycle routes, and PDR in a balloon-based testbed [6]. Focusing on the urban scenario, which aligns most closely with our study, several limitations can be identified. First, the number of measurement points and the covered distances are relatively small (only four points and up to 1.5 km, respectively). Additionally, the urban measurements were not conducted outdoors: the EDs were installed indoors, near windows, and connected to power sources to allow for extended operation. Second, in the mobility test, RSSI values are only reported for DR0, DR8, and DR9, with no corresponding PDR measurements. Finally, the relationship between PDR and RSSI is not analyzed for either the static or mobility scenarios.

Finally, A. Delplace et al. evaluate RSSI and PDR in an urban environment [7]. Nonetheless, although the overarching objective of their study and our work is comparable, several key differences can be identified. First, the frequency bands and corresponding configurations differ: our work is based on the EU863–870 band, whereas their study focuses on the US902-928 band, where the LR-FHSS modulation is configured differently. For example, the LR-FHSS operating channel width is 1.523 MHz in the US band, while it is 137 kHz or 336 kHz in the EU band (depending on the DR). Second, the ED transmission power in our setup is +14 dBm, the maximum permitted in the EU band, whereas it is +22 dBm in the US band. Third, the work by A. Delplace et al. considers only one LoRa DR (DR0 of the US band). In contrast, our study offers results for two LoRa DRs (DR0 and DR5 of the EU band), providing a finer level of granularity in the performance analysis. Finally, their work does not investigate the relationship between PDR and RSSI.

To the best of our knowledge, this article is the first that provides a comprehensive LR-FHSS performance study in urban and semi-urban scenarios, measuring both PDR and RSSI and showing their relationship at a greater number of distances between ED and GW in a variety of environments, and considering all EU band LR-FHSS DRs, while comparing their performance with LoRa DRs. As shown in Section 4, Section 5 and Section 6, the present study advances the state of the art and expands the knowledge in experimental LR-FHSS performance evaluation, illustrating the limits of LoRa when compared with LR-FHSS, and quantifying the behavior of all EU-band LR-FHSS DRs in the considered scenarios.

## 3. Background on LoRaWAN and LR-FHSS

**Figure 1:**
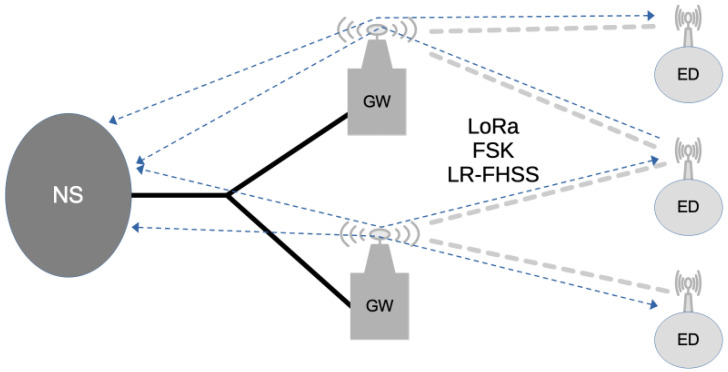
LoRaWAN network general architecture.

LoRaWAN defines an architecture and a communication protocol [28] suitable for devices deployed in an IoT network. This architecture includes three main elements (see Figure 1): Network Server (NS), GW, and ED. EDs exchange LoRaWAN frames with the NS. Each ED can reach the NS through a LoRaWAN link between them, with the help of one or more GWs. While a GW is connected to an NS through conventional IP-based backhaul connections (e.g., Ethernet, 4G or 5G mobile data network, etc.), EDs connect through GWs via wireless links based on LoRa, Frequency Shift Keying (FSK), or, more recently, the LR-FHSS modulation.

**Figure 2:**
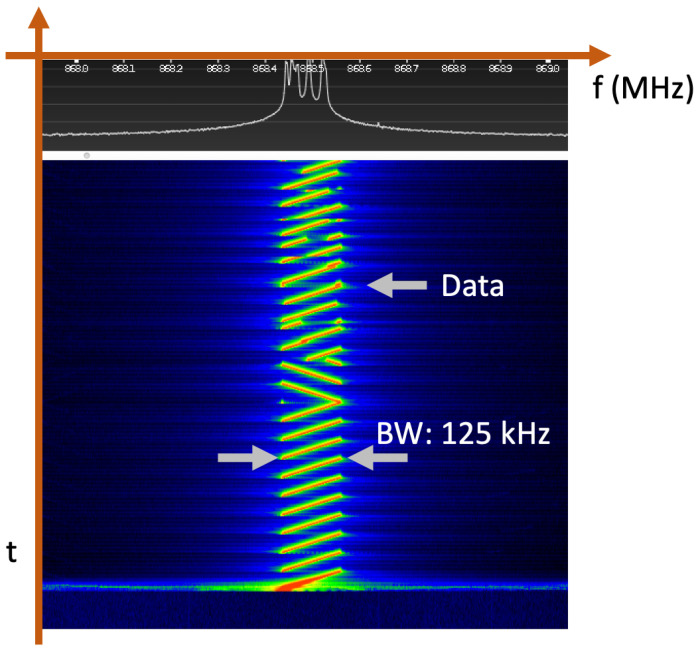
Experimental capture of the LoRa modulation behavior.

LoRa modulation [29] is based on CSS, where the signal frequency is progressively increased from the lower to the upper bandwidth limits (see Figure 2), usually 125 kHz, 250 kHz, or 500 kHz, depending on the radio frequency band plan. LoRa is characterized by strong immunity to interference and noise, an extended communication range, and a low power requirement for its low-bit-rate links. LoRa modulation utilizes orthogonal spreading factors (SFs), which enable the support of a greater number of devices within the system. Moreover, a higher SF provides more reliability and lower data rates (see Table 1). The greater processing gain given by LoRa compared to other modulation techniques has an impact on lower power requirements. LoRa modulation also includes an error correction function to increase transmission robustness.

LR-FHSS [8,30] is a new modulation scheme designed to improve network scalability and interference resilience compared to LoRa’s CSS modulation. Unlike LoRa, LR-FHSS uses frequency hopping across narrow sub-carriers to reduce collisions and enable support for a larger number of simultaneous uplink transmissions. Currently, LR-FHSS is used exclusively for uplink communication, while downlink transmissions continue to rely on standard LoRa modulation.

**Figure 3:**
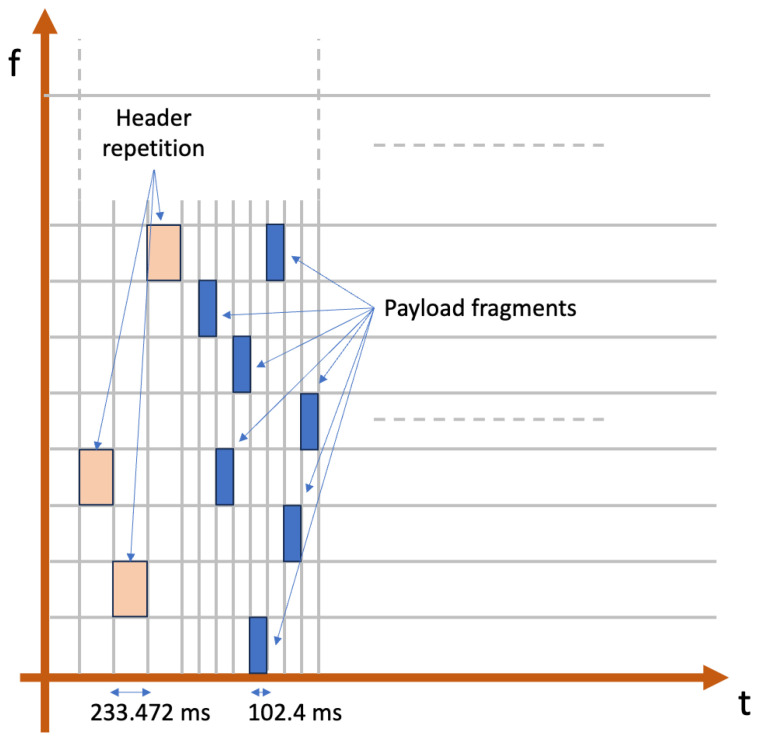
LR-FHSS data transmission, composed of the header repetitions and the fragmented payload.

LR-FHSS introduces additional steps during a transmission: when a physical packet is transmitted, 2 or 3 copies of the header are sent (depending on the DR), changing the sub-carrier frequency at each repetition, for increased redundancy and communication robustness. The transmission of a header has a duration of 233.472 ms. The remaining part of the packet is fragmented, transmitting each chunk on a different sub-carrier, which is changed every 102.4 ms (see Figure 3). Both header replicas and payload fragments are sent using a Gaussian Minimum-Shift Keying (GMSK) modulation, whereby one symbol carries one bit [31]. LR-FHSS also performs error correction based on convolutional coding, with coding rates (CRs) 1/3 and 2/3.

In the European (EU) 863-870 band, the LoRaWAN specification defines seven DRs based on LoRa modulation, one DR based on FSK, and four DRs based on LR-FHSS (see Table 1).

For DR0 to DR6, a specific SF is used, from SF12 to SF7, respectively. A higher SF value gives a lower bit rate and a greater transmission robustness. The resulting bit rates span from 250 bps to 11 kbps, respectively. DR7 uses FSK, offering a bit rate of 50 kbps. Note that only DR0 to DR5 are mandatory to be supported by an ED.

DR8 to DR11 use the new LR-FHSS modulation, applying a combination of two CR values, 2/3 and 1/3, a per-hop bandwidth of 488 Hz, and two different operating channel width values (137 kHz and 336 kHz), providing two bit rate options: 162 bps and 325 bps.

LoRa and LR-FHSS also differ in the physical frame format. The maximum physical payload size depends on the DR used, being 71 bytes for DR0 and 70 bytes for DR8 and DR9. On the other hand, DR9 and DR11 can carry 135 bytes, and the highest maximum physical payload is 262 bytes, which is supported by DR7.

Three classes of LoRaWAN EDs are defined: A, B, and C. Class A is mandatory, and the most common and power-efficient. It is based on an ALOHA-type communication protocol, where the ED initiates the message exchange with the NS. That is, the NS can only transmit to an ED after a transmission from the ED to the NS. Frame transmission from the ED to the NS can be confirmed or unconfirmed. In the confirmed mode, the NS has to confirm the reception by means of an Acknowledgment (ACK) packet sent back to the ED.

In order for an ED to operate in a LoRaWAN network, it has to be configured and activated. To this end, the LoRaWAN specification offers two options: Activation By Personalization (ABP) and Over-the-Air Activation (OTAA).

ABP is based on configuring the ED before it is deployed in the field. On the other hand, OTAA uses a dynamic configuration process based on an initial exchange of control information between the ED and the NS, named the Join procedure. This process starts with the transmission of a Join-Request frame from the ED, which has to be answered by the NS with a Join-Accept frame. After a successful Join procedure, the ED gets a network identifier, an ED address, and keys used for encryption and authentication. Moreover, the ED can also receive configuration settings, such as additional radio frequency channels, the transmission power, or the DR to use, among others.

## 4. Experimental Evaluation

This section presents an in-depth evaluation of the performance of both LoRa and LR-FHSS under real-world conditions, more specifically, in urban and semi-urban scenarios. The analysis focuses on an outdoor measurement campaign designed to assess how these technologies behave over various distances between ED and GW, and varying signal propagation environments. The section begins by describing the experimental setup and follows with the experimental results obtained, considering several ED locations along four distinct paths from a GW, focusing on PDR and RSSI, and providing a qualitative discussion of the measured performance along each path. Then, it features a broader interpretation of the measurements through an aggregate analysis, offering a view of how LR-FHSS and LoRa behave regardless of the specific features of each environment. The section concludes with a discussion on the trade-off between link range and latency and energy consumption of the LoRaWAN DRs. As a side contribution of this article, the data collected and used in this study are publicly available (see the corresponding link at the end of the document).

### 4.1. Experimental Setup

In this study, we conducted PDR and RSSI measurements from unconfirmed packet transmissions performed by the ED, for all LR-FHSS DRs, and for DR0 and DR5 (i.e., the lowest and highest mandatory LoRa DRs in the EU band) on a fully operational LoRaWAN network, comprising an ED, a GW, and an NS, all of which support LR-FHSS. For the ED, we used Semtech’s LR1121DVK1TBKS development kit [32], which combines a Nucleo L476RG board with an LR-FHSS-capable radio module based on the LR1121 chipset [33]. The GW is a Kerlink Wirnet iBTS Compact that supports LR-FHSS after a firmware upgrade [34]. For the NS, we run Chirpstack version 4.11.0 [35], connected to the same Ethernet LAN as the GW.

**Figure 4:**
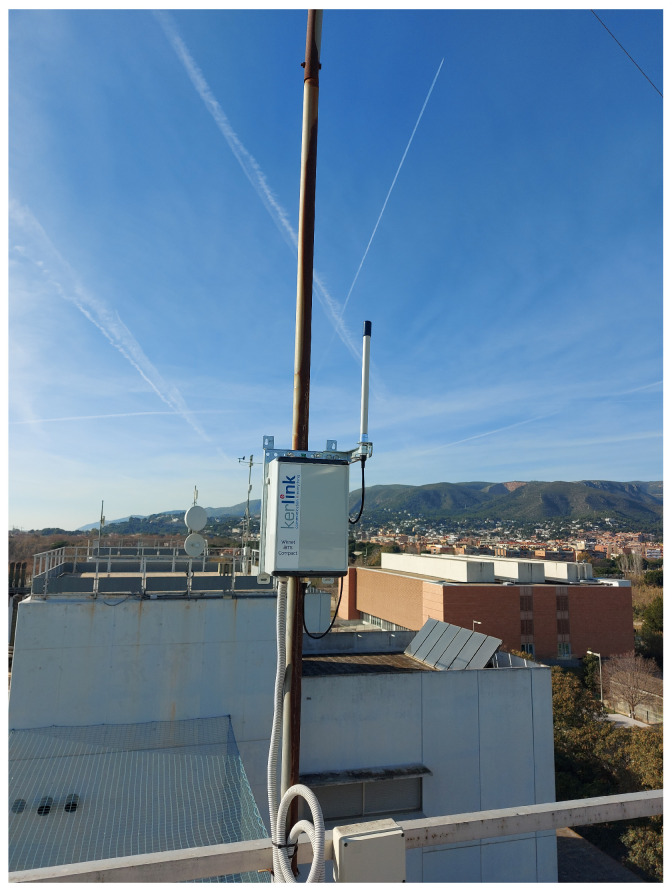
GW installed on the rooftop of one of the university buildings.

For the tests, the GW was placed on the rooftop of one of the Escola d’Enginyeria de Telecomunicació i Aeroespacial de Castelldefels (EETAC)’s university buildings, at a height of approximately 25 m, without nearby obstructions (cf. Figure 4). The ED’s transmit power was set to +14 dBm, and the GW’s transmit power to +27 dBm, which are the maximum allowed for the EU868 band [4]. The application-layer payload of each transmitted packet had a size of 1 byte. These settings are chosen to maximize the link range. For every combination of ED location and DR used, the number of transmitted packets exceeded 200. The RSSI value is only measured and stored for packets that have been correctly received and decoded. As a result, the available RSSI statistics are biased toward higher signal levels and can inaccurately reflect the actual average channel quality or signal conditions experienced by the device. This bias can lead to an overestimation of link quality, especially when significant packet loss occurs.

### 4.2. Scenarios

**Figure 5:**
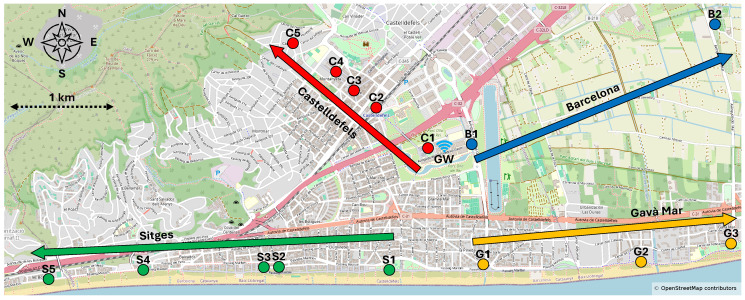
Castelldefels area map showing the considered measurement points and path directions.

We performed measurement campaigns, placing the ED in different locations along four directions starting from the GW. This methodology enables a systematic assessment of how LoRa and LR-FHSS perform under heterogeneous environmental and signal propagation conditions, such as line-of-sight (LOS), non-line-of-sight (NLOS), and mixed scenarios. Figure 5 shows a map with the different measurement points and overlying path directions, towards the cities and areas of Castelldefels, Sitges, Gavà Mar, and Barcelona. At each measurement point, all DRs were tested sequentially, during the same time frame and on the same day.

Table 2 shows the geodesic distances between the GW and the ED locations, as well as ED elevations, organized by direction. Distances range from hundreds of meters to 4 km. We next provide further details and the experimental results corresponding to each one of the four directions considered.

#### 4.2.1. Towards Castelldefels

The first set of outdoor measurements (locations C1-C5) was conducted in the uphill direction from EETAC toward the center of Castelldefels and beyond (northwestward). The urban environment of Castelldefels features low- to mid-rise residential buildings, open street layouts, and moderate vegetation density, providing generally favorable conditions for wireless signal propagation.

**Figure 6:**
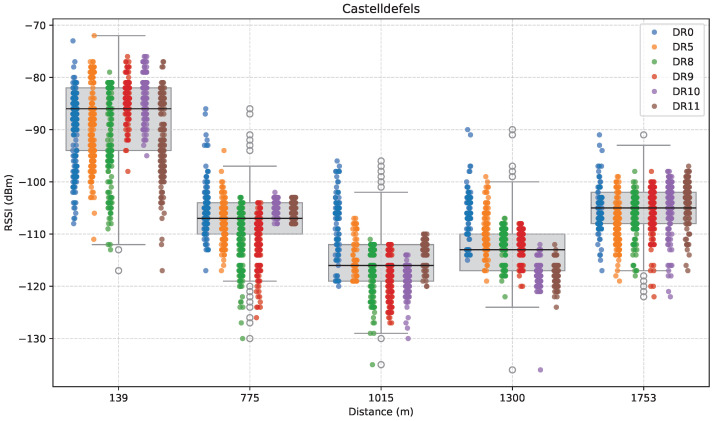
RSSI versus distance in the Castelldefels direction.

**Figure 7:**
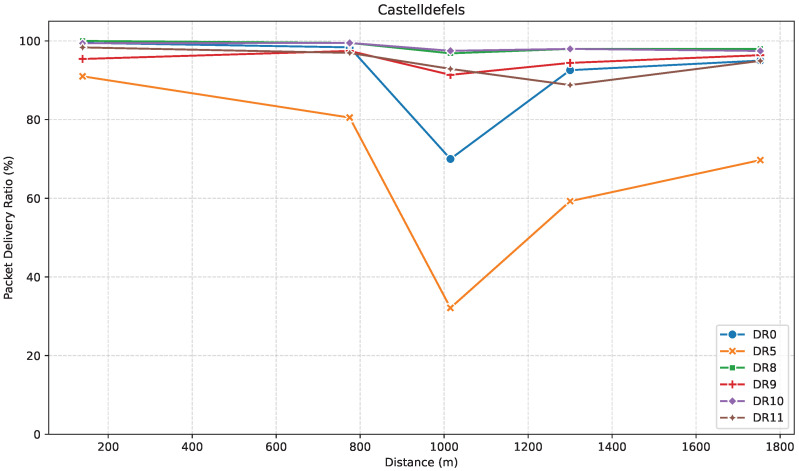
PDR versus distance in the Castelldefels direction.

**Figure 8:**
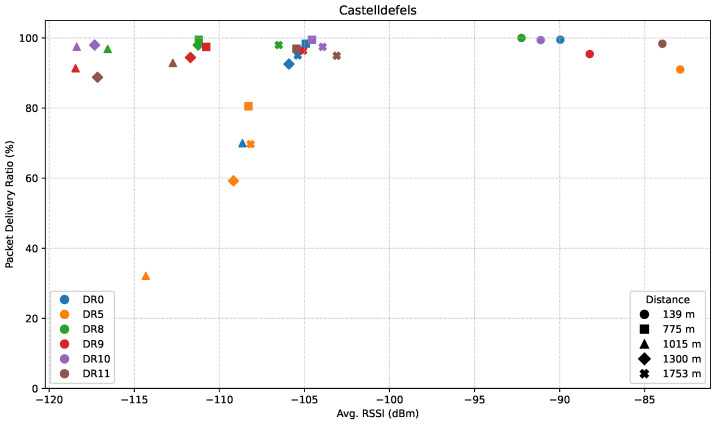
PDR versus average RSSI in the Castelldefels direction.

The results of this measurement set are shown in Figure 6, Figure 7 and Figure 8. First, Figure 6 displays RSSI as a function of distance along the measurement path. It is represented by a boxplot that summarizes the distribution of RSSI values at each location, with an overlaid stripplot indicating the individual data points, color-coded by DR.

As shown in the figure, at 139 m (C1), although presenting the greatest variability, RSSI remains high in all cases (with a median value of approximately −86 dBm), as we expected due to the proximity to the GW. Further away, at 775 m (C2), which is located just behind a train station, RSSI decreases overall to a median of around −107 dBm, particularly for DR8 and DR9, reflecting both increased distance and the influence of local obstructions. The significant RSSI variance observed for DR8 and DR9 was attributed to the time-variable passenger density nearby.

Subsequent measurements at 1015 m (C3), located in a centric square, revealed another drop in the overall RSSI performance to around −116 dBm, mainly due to the increased distance and obstacles that contribute to fading. At 1.3 km (C4), despite the greater distance, the median RSSI improved to approximately −114 dBm. The greater RSSI values at this location were likely due to a slight elevation increase, improving the signal strength by increasing Fresnel zone clearance and reducing obstruction and multipath effects in NLoS conditions, as shown by a greater average RSSI.

At the farthest location (C5, 1753 m), the median RSSI of successfully received packets improved again to approximately −105 dBm. Despite the distance increase, the greater elevation improved the communication conditions.

PDR is a key metric to assess communication reliability. Figure 7 illustrates how PDR evolves with distance for the DRs considered, in this direction.

At C1, PDR remained higher than 90% in all cases. Then, at C2, while the majority of DRs led to around 98% of PDR, DR5 experienced a significant drop to ~80%, most likely due to its lower robustness.

At C3, PDR dropped steeply, this time especially for DR5 to around 32%, but also for DR0 to ~70%. In this case, PDR for LR-FHSS remained mostly stable. Notably, DR0 outperformed DR9 in this environment, possibly due to the lower data rate of DR0 being more robust in noisy or obstructed conditions. A local minimum in PDR around C3 is apparent across almost all DRs. Beyond this point, higher elevation appears to restore or enhance link quality despite the increased distance.

At C4, both LoRa and LR-FHSS generally showed performance improvements. Specifically, the PDR for LoRa DR0 increased back to ~93% PDR, and reached 60% for DR5. At C5, while some RSSI values exceeded those at C2, the corresponding PDR did not necessarily improve, showing that a stronger signal does not always correlate with better link quality. Multipath propagation, shadowing, and other environmental effects may contribute to RSSI values in ways not directly linked to successful packet delivery.

Overall, LR-FHSS DRs (DR8–DR11) maintained PDRs above 85%, demonstrating robust performance even at longer ranges. Among them, DR8 and DR10 performed slightly better than DR9 and DR11, due to more robust coding rates and additional header repetitions that improve resilience under challenging conditions. LoRa DRs, in contrast, exhibited more variable behavior, with DR0 outperforming DR5, which aligns with its higher spreading factor and greater resilience to noise and attenuation.

Finally, Figure 8 illustrates the relationship between PDR and average RSSI for the DRs considered, in the Castelldefels direction. A general trend is observed in which higher RSSI values tend to correspond to higher PDR, whereas only LR-FHSS maintains high reliability at lower RSSI levels.

LR-FHSS DRs consistently achieve PDR values exceeding approximately 90% even at relatively low RSSI levels. Their ability to maintain high reliability under degraded signal conditions suggests that the underlying modulation and coding schemes are particularly resilient to noise and attenuation, making them well-suited for long-range communications. In contrast, DR5 exhibits a steep decline in PDR as RSSI decreases: while DR8 and DR10 achieve nearly 100% PDR at –117 dBm, DR5 only reaches around 60% even at –109 dBm. DR0 remains more stable, with PDR values ranging from approximately 70% to 98%, but it does not achieve the same level of consistency as the LR-FHSS configurations (DR8–DR11), which maintain high PDR even at lower RSSI values.

#### 4.2.2. Towards Sitges

The second set of outdoor measurements (S1-S5) was carried out following the coastal promenade heading to Sitges (westward). This path runs below the elevation of the university rooftop, where the GW is installed. It is important to note that, although the path is generally characterized by open spaces, there is no direct LoS between the receiver and most of the measurement points along this route. Along the promenade, various vegetation clusters and residential buildings obstruct the path, hampering signal propagation.

**Figure 9:**
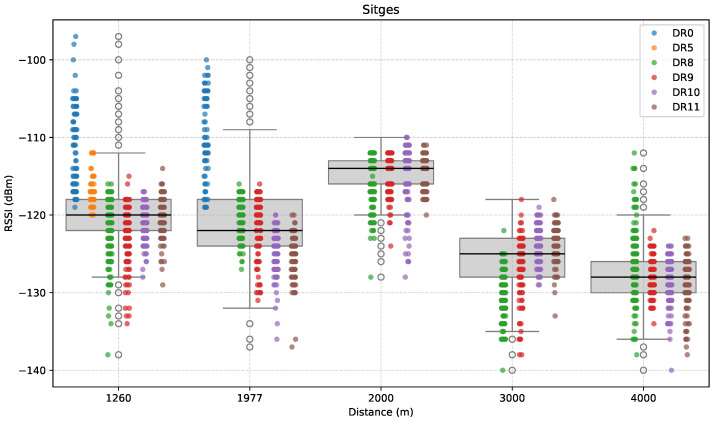
RSSI versus distance in the Sitges direction.

**Figure 10:**
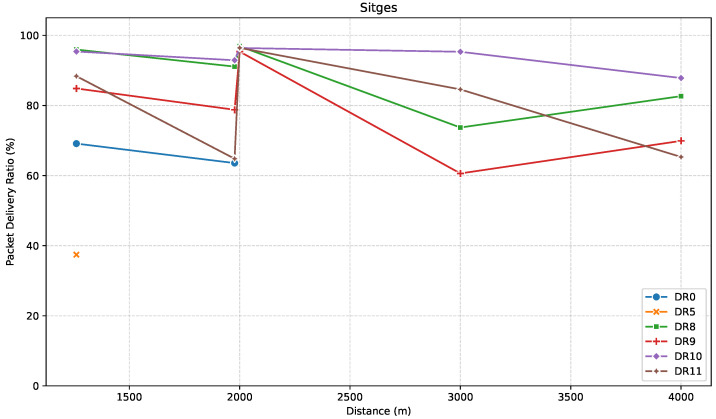
PDR versus distance in the Sitges direction.

**Figure 11:**
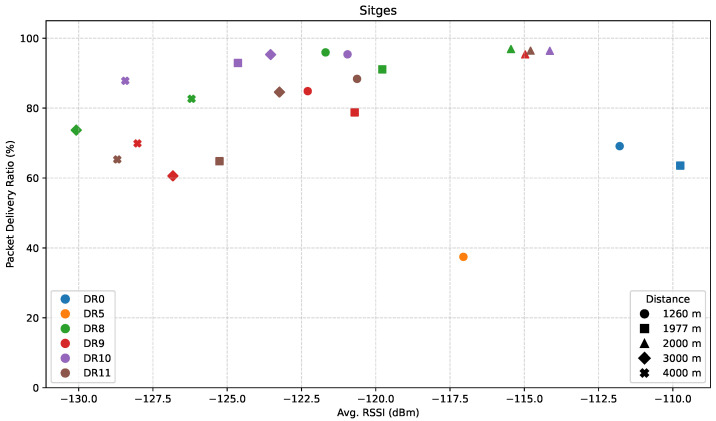
PDR versus average RSSI in the Sitges direction.

Figure 9, Figure 10 and Figure 11 depict the results for this direction. First, Figure 9 presents the RSSI measurements as a function of distance along the promenade. Notably, the distribution of RSSI values across DRs and distances shows that RSSI is influenced not only by distance, but also by environmental characteristics.

The first test point was at a distance of 1260 m from the GW (S1). While both LoRa DR0 and DR5 remained operational at this point, performance had already degraded noticeably in comparison with the Castelldefels direction. Concretely, the median RSSI of successfully received packets was −120 dBm at this point, as opposed to around −114 dBm in C4, where the distance between ED and GW is similar.

Then, we attempted to measure at 2 km. However, at this location, LoRa connectivity, even at its most robust configuration (i.e., DR0), was no longer possible. As a result, an intermediate measurement was conducted at 1977 m (S2) to identify LoRa’s coverage limit in that direction. In this case, the median RSSI expectedly dropped (to around −123 dBm), mainly due to the increased distance.

The following test location was at 2 km (S3), where the median RSSI increased to around −114 dBm, instead of decreasing with distance. This deviation from the expected monotonic RSSI decay strongly suggests the influence of local environmental factors, such as improved LoS (i.e., increased Fresnel zone clearance) and reduced obstruction at this location. Interestingly, this anomaly coincides precisely with the last point at which LoRa DRs were no longer available.

Beyond the 2 km mark, only LR-FHSS DRs were evaluated, as LoRa was no longer able to maintain communication. At 3 km (S4), the median RSSI dropped even further than in S2 to around −125 dBm, due to the increasing distance, and dropped again to approximately −128 dBm at the farthest point of 4 km (S5).

Overall, signal strength generally decreased with increasing distance, as the median RSSI dropped from approximately −120 dBm at 1260 m to around −128 dBm at 4 km. However, a notable anomaly was the one recorded at 2 km (S3), where signal strength appeared stronger than at the previous locations.

Regarding RSSI variability, all measurement points exhibit wide interquartile ranges and extended whiskers. The spread of values, sometimes spanning 20–30 dB at a single location, reflects common real-world conditions such as multipath propagation and a changing environment.

To assess communication reliability across the locations in the Sitges direction, Figure 10 shows the PDR performance of the tested DRs as a function of distance.

At S1, PDR remained approximately around 90% for the LR-FHSS DRs, but the LoRa ones showed significantly different behavior. For DR0, PDR dropped to around 70%, whereas, for DR5, PDR showed a value of just 37%, indicating that it was nearing the edge of its effective range. At S2, DR5 did not achieve connectivity, but DR0 still showed a PDR of around 64%, a result that was higher than expected considering that DR0 connectivity was lost just a few meters farther in S3. This behavior illustrates the non-steady and environment-dependent nature of LoRa coverage. At S2, PDR values for the LR-FHSS DRs slightly decreased, as expected, due to the increased distance. The only exception was DR11, with a significant PDR decrease to around 65%.

In the third location, S3, only LR-FHSS DRs had connectivity, and the PDR instead increased to a maximum of around 95% for DR8-DR11. This coincides with the unexpected increase in median RSSI that we identified in Figure 9 at the 2 km point, reaffirming the local trend of better connectivity. Measurements at the more distant S4 and S5 showed that all four LR-FHSS DRs (DR8-DR11) still provided reliable connectivity. This confirmed that, while LoRa failed to achieve connectivity beyond ~2 km in this direction, LR-FHSS maintained effective performance even at double the distance.

Figure 11 illustrates the relationship between the PDR and the average RSSI for this measurement path.

Compared to the Castelldefels direction, the data points in this scenario are noticeably shifted toward lower RSSI values (ranging from approximately −130 dBm to −110 dBm), indicating a generally more attenuated signal environment. Despite the weaker signal conditions, DR8 and DR10 consistently demonstrate excellent performance. These configurations maintain PDR values above 90% across a broad RSSI range, extending down to approximately −122 dBm. This high degree of reliability under adverse propagation conditions further reinforces their suitability for long-range and low-RSSI scenarios.

Conversely, DR9 and DR11 exhibit greater variability in performance. While they remain generally functional, their PDR fluctuates between approximately 60% and 95%, suggesting a moderate decline in robustness compared to DR8 and DR10.

Among the LoRa DRs, DR0 is only present within the highest RSSI range in the dataset (−112 to −110 dBm), where it achieves a moderate PDR of around 60–70%. It does not appear at lower signal levels, implying that its coverage limit was reached earlier in this direction. DR5 performs particularly poorly, with a single data point registering a PDR below 40% at an average RSSI of −117 dBm, showing vulnerability to degradation in weak signal environments.

#### 4.2.3. Towards Gavà Mar

The third outdoor measurement path (G1–G3) stretched toward Gavà Mar (eastward), as shown in Figure 5. This path runs below the elevation of the university building rooftop, where the GW is installed. Also, there is significant vegetation density and limited LoS due to natural and built constructions.

**Figure 12:**
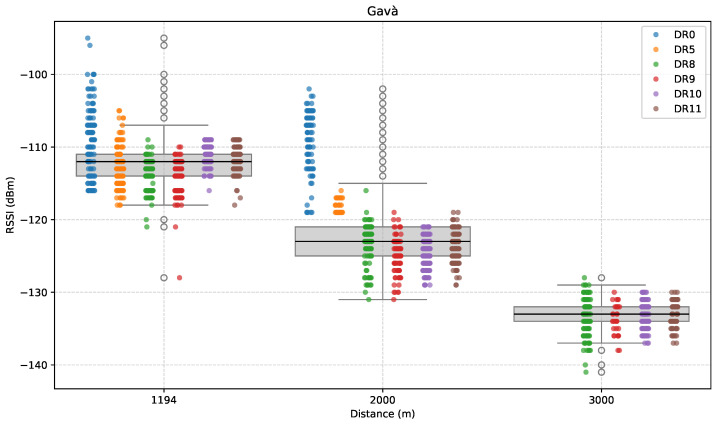
RSSI versus distance in the Gavà Mar direction.

**Figure 13:**
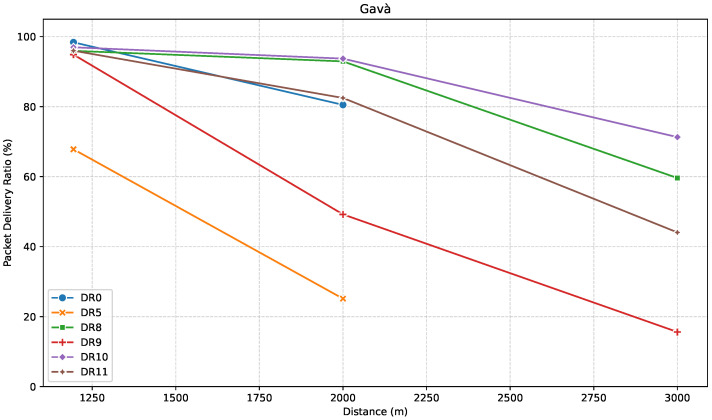
PDR versus distance in the Gavà Mar direction.

**Figure 14:**
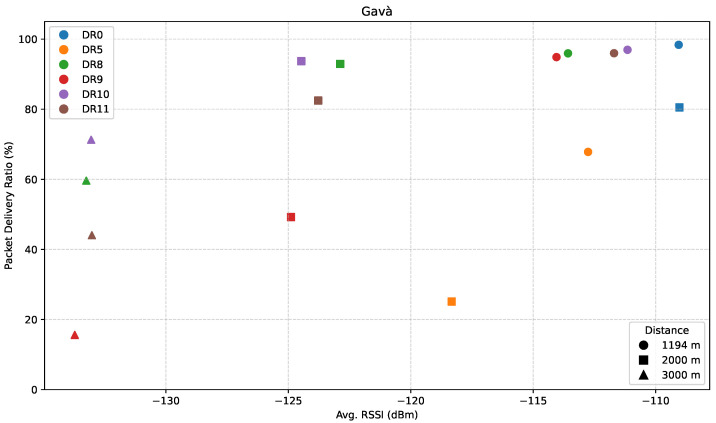
PDR versus average RSSI in the Gavà Mar direction.

Figure 12, Figure 13 and Figure 14 show the results for this specific direction. First, Figure 12 presents the evolution of RSSI with increasing distance along the path.

In the first test point (G1), located approximately 1.2 km from the GW, all DRs, both LoRa and LR-FHSS, were able to establish communication. Specifically, we measured a median RSSI across DRs of approximately −112 dBm, which is mostly greater than for similar distances in previous scenarios. The next measurement was conducted at 2 km (G2), with the goal of exploring the upper limit of LoRa coverage in this direction. As expected, performance degraded compared to the previous point, to a median RSSI of successfully received packets of approximately −123 dBm.

The final test point (G3), located at 3 km, marked the effective limit of LoRa coverage in this direction. The device was unable to complete the Join procedure, even after trying multiple nearby locations. Consequently, measurements at this distance were conducted exclusively using LR-FHSS, which continued to function, albeit with noticeable RSSI degradation compared to the 2 km point. Specifically, we measured a median RSSI of around −133 dBm.

In contrast to the anomalies noted in the Castelldefels and Sitges directions, the Gavà Mar path exhibits a decreasing attenuation profile. The smooth degradation suggests a propagation environment where signal loss is primarily governed by distance, with no significant anomalies in terrain or LoS conditions along the route. LoRa DRs did not have connectivity at G3, while LR-FHSS remained operational, confirming its superior resilience compared to conventional LoRa in challenging propagation environments.

It is worth mentioning that RSSI variability remains significant. At each location, wide interquartile ranges and extended whiskers indicate fluctuations of up to 20 dB.

Figure 13 displays the PDR performance for the ED location points along this route.

Results showed that, at G1, DR0 surprisingly achieved the highest PDR (~99%), outperforming even the most robust DRs of LR-FHSS. The rest of the results followed expected trends, with DR5 showing the weakest reliability, and the relative behavior among LR-FHSS DRs (DR10 and DR8 outperforming DR11 and DR9, respectively) remaining consistent with prior observations.

At G2, while LoRa DR0 still achieved a respectable PDR of 80%, DR5 and DR9 performed significantly worse, particularly DR9, which showed an unexpectedly low PDR of around 50%. The reason for this deviation could not be conclusively determined. However, it illustrates how the variability of real-world conditions may affect communication performance.

Finally, at the farthest point (G3), it is important to highlight that the distributions of RSSI values across all LR-FHSS DRs were nearly identical, yet the corresponding PDR values varied significantly, ranging from 15% to 71%.

A distinctive feature of this direction is the steady decline in link quality observed across all DRs. At G2, LoRa DR5 already drops to a PDR of approximately 25%, signaling it is close to its coverage limits. DR0, by contrast, still achieves around 80%, which is unexpectedly high considering that it completely disappears in G3. This suggests a sudden and sharp degradation in the environment beyond that point. Even LR-FHSS DRs show performance degradation in this scenario. DR9 is the most affected, falling below 20% PDR at 3 km, while DR11 drops to around 45%. Even the more robust DR8 and DR10 experience a significant PDR reduction, albeit to a lower degree. These results explain the limited coverage potential in this direction, even for LR-FHSS, highlighting the impact that specific paths can have on system performance.

Finally, Figure 14 presents the relationship between PDR and average RSSI for the path towards Gavà Mar.

In contrast to the previous directions, this environment appears particularly challenging, with average RSSI values as low as −132 dBm. The figure illustrates a clear stratification in performance between modulation schemes under such adverse conditions. As observed in previous directions, DR10 and DR8 once again emerge as the most robust settings, maintaining PDRs above 90% even at weak RSSI levels. However, their performance begins to degrade when the distance increases to 3 km.

DR11 demonstrates moderate reliability, achieving PDR values close to 97% at stronger signal levels, but its robustness decreases sharply with lower RSSI. Under the same conditions, DR9 exhibits significantly poorer performance, with a PDR dropping as low as 15%. This suggests it is particularly vulnerable to signal attenuation and unsuitable for operation in such long-range, obstructed paths.

DR0 achieves a high PDR (above 80%), but it is only recorded within the strongest portion of the RSSI range, and it is absent on the weaker side, confirming its limited coverage. DR5 remains the least robust configuration, with PDR values consistently low (~25% to ~68%), confirming its limited practical value in outdoor deployments under weak signal conditions.

#### 4.2.4. Towards Barcelona

The fourth and final outdoor measurement (B1, B2) path followed the direction toward Barcelona (northeastward), as shown in Figure 5. In this direction, the area presents mostly unobstructed conditions, with sparse, low-rise buildings and few significant obstacles to signal propagation. However, vegetation is quite dense due to the presence of farmland.

**Figure 15:**
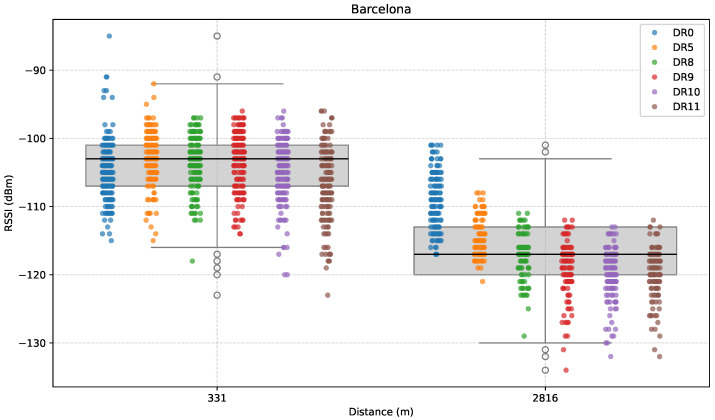
RSSI versus distance in the Barcelona direction.

**Figure 16:**
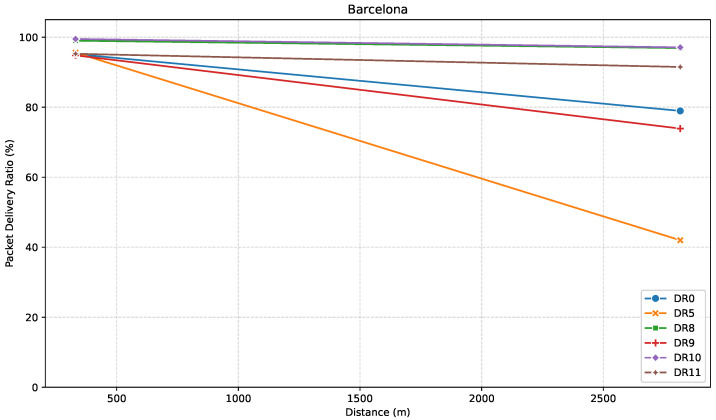
PDR versus distance in the Barcelona direction.

**Figure 17:**
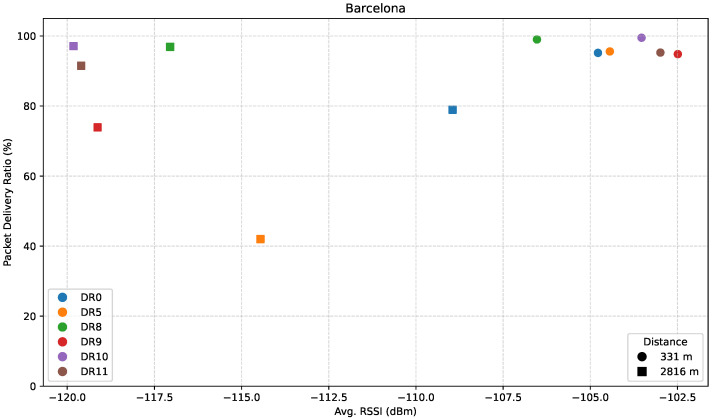
PDR versus average RSSI in the Barcelona direction.

Figure 15, Figure 16 and Figure 17 show the results for this direction. To begin with, Figure 15 shows how RSSI values vary across the considered distances.

In the first test point (B1), results were as expected for a distance of hundreds of meters, and without significant obstacles: a notable median RSSI of successfully received packets of around −104 dBm. At the second test point (B2), RSSI takes an expected drop to −116 dBm due to the significantly increased distance, plus the effect of dense vegetation between ED and GW.

Overall, as expected, RSSI values tend to decrease with distance. Although variability in the results is around 25 dB, the difference between median RSSI values is around 14 dB, which is not that large considering a 2.5 km distance difference. This could be explained by the LoS characteristics of the path.

Figure 16 illustrates PDR over distance. Results show that at B1, LR-FHSS performs the best overall, with DR10 reaching nearly 99.5% PDR and DR8 not far behind, at 99%. The rest of the DRs provide slightly lower, but still high, and very similar PDR values. Interestingly, LoRa DR5 slightly outperforms DR0, which is unusual given the typically better performance of DR0.

Further away, at B2, performance differences become much clearer. LoRa DRs are noticeably affected, with DR5 dropping to a PDR of just 42% and DR0 achieving around 79%. On the other hand, LR-FHSS DRs perform much more reliably. DR10 maintains excellent performance with a PDR of 97%, and DR8 follows closely at 97%. Remarkably, while DR9 achieves only 74%, it still outperforms LoRa DR5 but falls behind DR0. DR11 performs better than DR9, reaching a PDR of 91%, which may be attributed to its use of a greater number of hopping channels, even though it shares the same modulation settings as DR9.

Figure 17 presents the relationship between PDR and average RSSI for the path towards Barcelona.

Results show that for B1, all points yield both a good average RSSI of around −104 dBm, and a PDR near 100%. For the farthest point, B2, however, despite a significant drop in signal strength of more than 10 dB in most cases, LR-FHSS DRs continue to deliver strong PDRs, especially DR8, DR10, and DR11, which further validates their suitability for long-range communication.

### 4.3. Aggregate Performance Analysis

This subsection presents an analysis of the aggregate performance of LoRa and LR-FHSS in terms of RSSI, path loss, and PDR as a function of distance, and PDR as a function of RSSI, based on the gathered field measurements from all scenarios. This analysis allows us to study the results regardless of individual directions, see how well each DR handles weak signals, and how consistent the connectivity is at long distances.

#### 4.3.1. Aggregate RSSI Versus Distance

**Figure 18:**
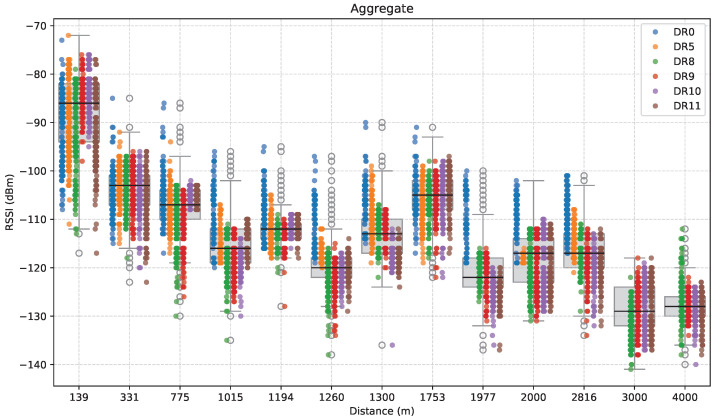
Aggregate RSSI measurements as a function of distance.

Figure 18 presents the aggregate RSSI measurements as a function of distance, combining all obtained data regardless of direction or path.

As expected, the median RSSI of successfully received packets follows a generally decreasing trend with distance, although with fluctuations, illustrating typical path loss behavior in realistic wireless communication environments. There is also high dispersion of values observed at each distance, which highlights the influence of environmental variability on the measured RSSI values. Although distance is a dominant factor, conditions such as obstructions, elevation, and multipath propagation have a significant impact on the received signal power.

Some ED locations stand out from the general trend. For example, at 1753 m (and similarly at 1300 m), the median RSSI is notably higher than at some of the shorter distances. These deviations, already observed in the path-specific plots, are consistent with the improved visibility between the ED and the GW at the corresponding locations. On the other hand, LoRa DRs lose connectivity beyond ~3 km, while only the LR-FHSS ones remain operational.

**Figure 19:**
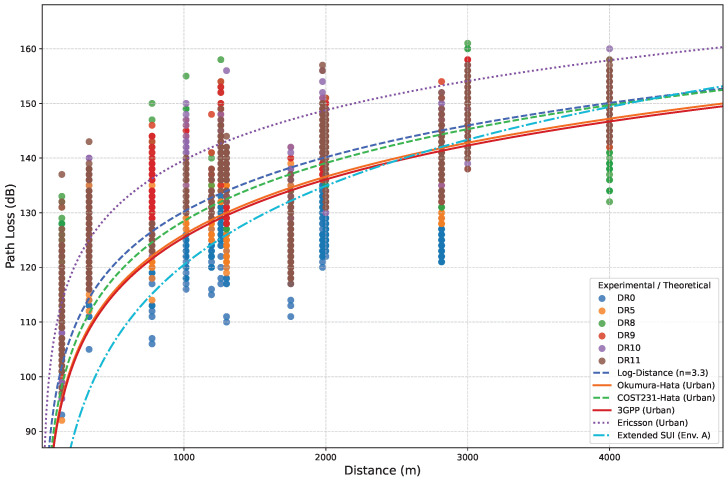
Comparison of experimental and theoretical path loss as a function of distance.

To thoroughly characterize the experimental results, Figure 19 compares the measured path loss with several prevalent theoretical propagation models for LPWAN technologies, adapted to urban environments [36]. The models considered include Okumura-Hata, COST231-Hata, 3GPP, Ericsson, extended Stanford University Interim (SUI), and the standard log-distance model. For the log-distance model, we fitted the propagation coefficient by testing different values of *n* and selecting *n* = 3.3, which yielded the lowest Mean Squared Error (MSE) with respect to our empirical measurements. Note that the selected path-loss exponent value (*n* = 3.3) corresponds to a shadowed urban environment, characterized by predominantly LoS propagation with intermittent obstructions (e.g., buildings and other structures) [37]. This aligns with the conditions of our experimental environment, as detailed in Section 4.2.1, Section 4.2.2, Section 4.2.3 and Section 4.2.4.

The Ericsson and the extended SUI models poorly predict the behavior of the presented scenarios, resulting in the highest MSE values. The Ericsson propagation model yields an overestimation of path loss due to its inherent calibration for high-density urban settings. Furthermore, while the extended SUI model in its environment A variation demonstrates the best performance among the three (A, B, and C) due to its generalized characteristics for hilly, dense foliage terrain, it is still not appropriate to accurately model our test environment, which consists of a mixed urban, suburban, and coastal landscape. Conversely, the remaining four models provide more accurate path loss predictions, particularly for distances close to 1000 m and beyond. Among them, the fitted log-distance model achieves the lowest MSE, closely followed by the COST231-Hata model. This result indicates that the calibrated log-distance model offers the most accurate mathematical representation of the propagation conditions observed in our specific experimental scenarios.

#### 4.3.2. Aggregate PDR Versus Distance

**Figure 20:**
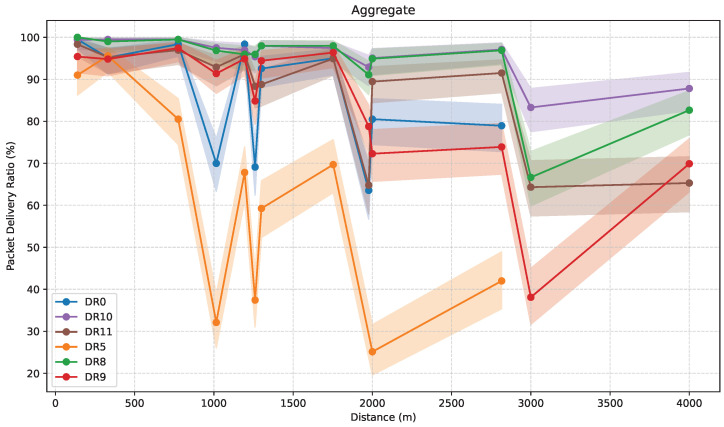
Aggregate PDR measurements as a function of distance, with 95% confidence intervals.

Figure 20 shows the aggregate PDR as a function of distance, for all measurement paths and DRs considered, with 95% confidence intervals. This plot provides a global overview of the communication reliability over varying distances in all the scenarios in this study.

PDR tends to decrease with distance, reflecting the weakening signal strength observed in Figure 18. However, PDR strongly depends on the specific location associated with each considered distance and the configuration used. Specifically, 1300 m and 1753 m locations present a PDR increase that deviates from the general inverse relationship between distance and signal quality. These localized performance improvements are directly attributed to enhanced LoS visibility between the ED and the GW, confirming that path-specific characteristics, rather than distance alone, influence data delivery success. The performance gap between different DRs becomes particularly evident beyond 2000 m, where some configurations maintain high delivery rates while others exhibit significant degradation. The observed results allow for the classification of the tested DRs into three distinct tiers: DR10 and DR8 (high reliability), DR11 and DR9 (medium reliability), and DR0 and DR5 (low reliability).

In the high-performance tier, DR10 exhibits the highest reliability across all tested distances. It maintains a PDR above 95% up to 2000 m, and its PDR remains above 85% even at 4000 m. DR8 performs comparably to DR10 at short and medium ranges. Although its performance dips around 3000 m, it recovers to exceed 80% PDR at 4000 m, indicating its suitability for extended coverage. DR10 delivers the most robust long-range performance, not only due to its lower transmission speed and stronger coding rate and redundancy, but also likely because it utilizes a wider range of frequency sub-carriers, compared to DR8.

In the medium-performance tier, DR11 delivers high reliability up to approximately 2816 m, after which its PDR declines sharply, reaching around 65% at 4000 m. This makes it a suitable choice for mid-range deployments, though it is less effective for edge-of-network coverage. DR9, in contrast, maintains stable performance at short distances but exhibits a marked decrease in PDR at 3000 m, dropping below 40%. Furthermore, the variability of results increases substantially, suggesting inconsistent performance. Overall, the results indicate that higher transmission bit rate and lower transmission redundancy reduce robustness, compared with DR10 and DR8, although this effect is less pronounced in DR11 due to its larger number of usable sub-carriers.

Finally, in the low-performance tier, DR0 demonstrates relatively good performance at short distances; however, it fails to maintain connectivity at ~3 km and beyond, indicating its limited suitability for long-distance communication. DR5 exhibits the lowest overall reliability, with PDR values declining rapidly and often falling below 40% even at moderate distances. Consequently, this DR is unsuitable for applications requiring high reliability over medium to long ranges.

The results highlight the critical role of DR selection in network design, especially in scenarios involving long-range communication or challenging environmental conditions. The performance differences among DRs are not solely attributable to transmission bit rate, since DR0 operates at a lower data rate than DR9 or DR11, but also to the underlying modulation scheme (LoRa or LR-FHSS), and the corresponding transmission redundancy.

#### 4.3.3. PDR Versus RSSI

**Figure 21:**
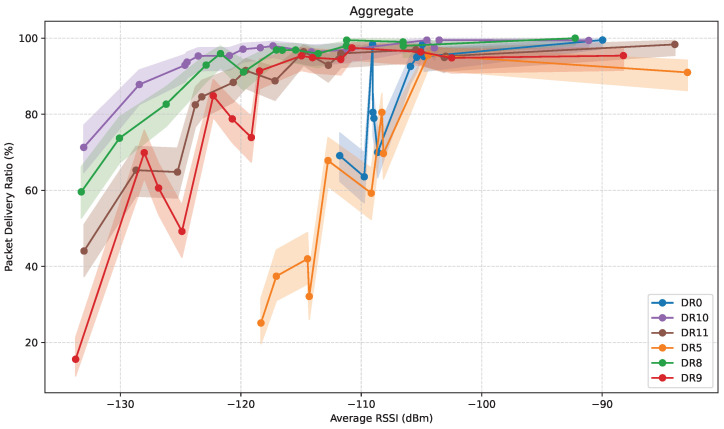
Aggregate PDR as a function of the average RSSI, with 95% confidence intervals.

Figure 21 illustrates PDR as a function of the average RSSI, for all DRs considered, with 95% confidence intervals. This figure shows how each DR performs in terms of average RSSI, regardless of the specific propagation phenomena that produce the corresponding RSSI values.

A clear pattern emerges across all DRs: assuming an initial high RSSI value, as the average RSSI decreases, the PDR remains consistently high until a specific threshold is reached. Beyond this point, the PDR drops noticeably. This phenomenon represents the sensitivity limit of each DR. The exact value of this threshold varies significantly across the tested DRs. The PDR degradation with decreasing RSSI is more pronounced for less reliable DRs: the greatest slope occurs for DR0-DR5, it is lower for DR11-DR9, and the lowest is offered by DR8-DR10. It is noteworthy that said degradation is not monotonic over distance; particularly, at the average RSSI points that correspond to ED locations at 1300 m and 1753 m, PDR increases instead of steadily decaying. These localized PDR enhancements can be attributed to the increased ED-GW LoS visibility, which effectively mitigates detrimental channel impairments such as multipath fading and shadowing at these specific locations.

Among all configurations, DR10 demonstrates the greatest tolerance to weak signal conditions. It maintains a high PDR, even above 90%, at average RSSI values as low as −125 dBm. This behavior reflects its suitability for long-range communication and challenging propagation environments, where signal attenuation is substantial. DR8 shows a similar performance profile, maintaining high reliability well into the low average RSSI region, closely tracking DR10’s performance across the entire range.

DR11 also performs reliably, although its tolerance to signal degradation is slightly lower. PDR values remain high when the average RSSI is above approximately −120 dBm, but start to decrease noticeably below this threshold. While still suitable for most scenarios, DR11 may become less reliable in the weakest signal conditions encountered at the edge of coverage. In contrast, DR9 exhibits a more abrupt drop-off. While it performs well when the average RSSI is stronger than around −118 dBm, its reliability declines steeply beyond this point, resulting in significantly lower PDR values. This sharp transition limits its effectiveness in environments where consistent signal strength cannot be guaranteed.

DR5 performs poorly under weaker signal conditions. It displays reduced reliability even at moderate average RSSI levels, with visible fluctuations and a general tendency toward low PDR values for average RSSI values below −110 dBm. This suggests that DR5 lacks the robustness required for operation in many real-world scenarios, particularly those involving NLoS propagation or long distances.

### 4.4. Trade-Off: Link Range vs. Latency and Energy Consumption

**Figure 22:**
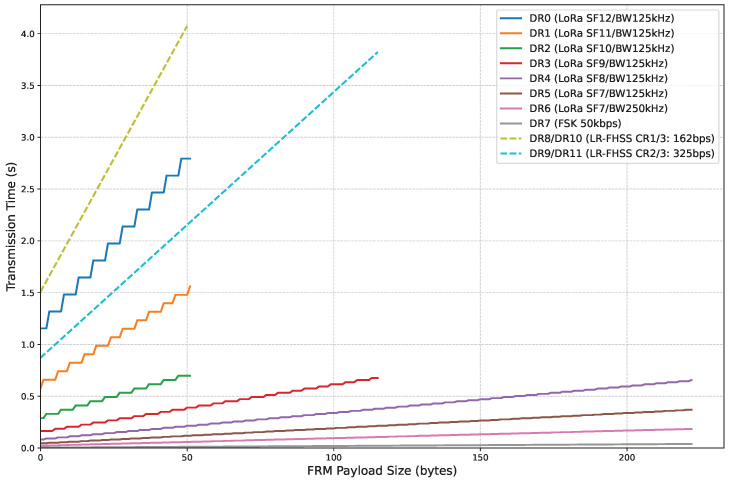
Frame transmission time as a function of FRM payload size and DR values.

A key design consideration in LoRaWAN is the inherent trade-off between communication parameters, often leading to a compromise among achievable link range, robustness, latency, and energy consumption. As demonstrated by the presented results, the LR-FHSS modulation scheme (utilizing DR8-DR11) is typically deployed to enhance both link range and robustness significantly. This benefit, however, frequently comes at the cost of increased latency and, consequently, higher energy consumption compared to standard LoRa DRs [5]. This trade-off is visually represented in Figure 22, which shows that DRs with a higher bit rate incur a shorter frame transmission time or Time-on-Air (ToA), whereas slower rates, including DR0 and the LR-FHSS DRs, exhibit a higher ToA for an identical FRM payload size.

An interesting consideration is that DR0 presents a notable exception to this typical compromise. DR0, which provides the longest link range among LoRa DRs, offers a shorter link range, but also greater ToA and energy consumption than DR9/DR11. This counterintuitive performance stems from the specific modulation, coding optimizations, and redundancy mechanisms inherent to the LR-FHSS modulation. These configurations not only provide a superior link budget but also result in a more efficient overall transmission than DR0.

## 5. Open Issues

While carrying out the study provided in this article, we identified a number of LR-FHSS issues and research gaps. This section aims to outline LR-FHSS open issues that may currently limit its applicability and performance, and/or might offer venues for future LR-FHSS evaluation, improvement, and standardization work.

### 5.1. Join with LoRa

The initial Join procedure represents a critical step in ED activation through OTAA (cf. Section 3). While LR-FHSS can improve uplink reliability, current LoRaWAN specifications define the Join request and response using LoRa modulation. This mismatch raises questions on why devices intending to operate with LR-FHSS must first rely on LoRa for OTAA-based network association, and how this dependency impacts coverage, network design, and scalability.

### 5.2. Downlink with LoRa

Even if uplink robustness and scalability are significantly enhanced via LR-FHSS, downlink transmission remains constrained to using the LoRa modulation. A key motivation for this choice could be the minimization of energy consumption at the EDs, since LoRa demodulation typically requires less complexity and shorter on-air times compared to LR-FHSS. However, this design decision introduces a trade-off: while energy efficiency is preserved, the potential benefits of LR-FHSS in downlink robustness and capacity remain untapped. For instance, if the response to an LR-FHSS frame, such as an ACK, is transmitted using LoRa, there is a non-negligible risk that the response will not be successfully received under adverse channel conditions. It remains an open question whether future revisions of LoRaWAN should support LR-FHSS-based reception windows, and under what circumstances such an adaptation would provide a net benefit for large-scale deployments.

### 5.3. Evaluation of LR-FHSS for NTNs

One of the motivating use cases of LR-FHSS is Non-Terrestrial Networks (NTNs), focusing on the use of the technology for Direct-to-Satellite IoT (DtS-IoT) connectivity with Low Earth Orbit (LEO) satellites. Therefore, an analysis of how well the current LR-FHSS is suited for NTN use cases would be essential. Specifically, evaluations should consider the greater propagation delays, high Doppler shifts, intermittent coverage, moving GWs (on board satellites) instead of fixed ones, and the impact of a high number of devices per GW, which are characteristics of NTNs. Ideally, the study should be carried out in collaboration with satellite IoT providers to conduct field tests. Additionally, the use of directional antennas and new maximum EIRP limits for NTN operation should be explored. A comprehensive evaluation of LR-FHSS robustness and adaptability under such demanding conditions is essential before its adoption for large-scale or global IoT connectivity.

## 6. Conclusions and Future Work

The main objective of this article was to evaluate the performance of LR-FHSS in real-world outdoor scenarios and to compare its behavior with conventional LoRa DRs (specifically DR0 and DR5). To do so, a measurement campaign was carried out using a dedicated setup with a GW placed on the rooftop of a university building in Castelldefels (near Barcelona), and an ED at different locations transmitting under varying configurations and propagation conditions. ED locations were determined within paths in four main directions. In each case, a detailed analysis of the PDR, RSSI, and the behavior of the DRs considered was conducted.

The results confirmed the enhanced robustness of LR-FHSS compared with LoRa, especially in NLoS and long-range conditions. While LoRa DR0 and DR5 showed rapid degradation of connectivity beyond certain distances, typically between 1.5 and 2 km, LR-FHSS DRs maintained high PDR values even up to 4 km, depending on the environmental conditions. This behavior was particularly evident in the direction towards Sitges, where LR-FHSS maintained above 90% PDR at 4 km, while LoRa was no longer operational beyond 2 km. The fundamental trade-off in LoRaWAN lies between link range and robustness on one hand, and latency and energy consumption on the other. Slower LoRa configurations extend range but increase ToA and energy usage. Interestingly, while the LR-FHSS modulation offers the greatest communication range overall, certain LR-FHSS configurations (DR9 and DR11) also achieve both lower ToA and reduced energy consumption compared to LoRa’s longest-range mode, DR0.

A noteworthy observation is that RSSI alone is not a sufficient predictor of link performance; in several cases, similar RSSI levels corresponded to significantly different PDR values. This suggests that other factors—such as multipath fading, or interference—may play a significant role. Nevertheless, when both the average RSSI and the DR are given, PDR can be reasonably predicted. To further characterize the specific propagation environment, a comparison between our experimental results and various theoretical propagation models was conducted. This analysis indicated that the log-distance model, when calibrated with a path loss exponent of *n* = 3.3, yielded the lowest MSE. Consequently, this model offers the most accurate characterization of the radio channel behavior within the measured scenarios.

From a practical perspective, this work has demonstrated the feasibility of deploying LR-FHSS in environments where conventional LoRa struggles, confirming its value for future LPWAN applications that require longer range or more reliable connectivity. The study has contributed with real-world performance evaluation results and a comprehensive analysis of a modulation scheme that, despite its inclusion in the LoRaWAN standard in 2020, remains underexplored in real environments, probably due to the challenges of setting up a LoRaWAN network with LR-FHSS support.

Finally, while this article focused on terrestrial deployments, we envision future studies investigating the performance of LR-FHSS in NTN scenarios.

## Figures and Tables

**Table 1:** DR definition for the EU863-870 band.

DR	Modulation	Spreading Factor	Coding Rate	Bandwidth (kHz)	Physical Bit Rate (bit/s)	Number of Header Copies	Number of Hopping Channels
0	LoRa	SF12	4/5 to 4/8	125	250	1	-
1	LoRa	SF11	4/5 to 4/8	125	440	1	-
2	LoRa	SF10	4/5 to 4/8	125	980	1	-
3	LoRa	SF9	4/5 to 4/8	125	1760	1	-
4	LoRa	SF8	4/5 to 4/8	125	3125	1	-
5	LoRa	SF7	4/5 to 4/8	125	5470	1	-
6	LoRa	SF7	4/5 to 4/8	250	11,000	1	-
7	FSK	-	-	-	50,000	1	-
8	LR-FHSS	-	1/3	137	162	3	35
9	LR-FHSS	-	2/3	137	365	2	35
10	LR-FHSS	-	1/3	336	162	3	86
11	LR-FHSS	-	2/3	336	365	2	86
12–14	RFU						
15	Defined in the specification [28]						

**Table 2:** Geodesic distances and elevation between the GW and the ED locations, organized by direction.

Direction	ED Location	Distance (m)	Elevation (m)
Castelldefels	C1	139	6.9
	C2	775	9.0
	C3	1015	11.0
	C4	1300	16.1
	C5	1753	56.2
Sitges	S1	1260	3.3
	S2	1977	10.1
	S3	2000	9.6
	S4	3000	9.1
	S5	4000	4.2
Gavà Mar	G1	1194	5.3
	G2	2000	7.8
	G3	3000	7.0
Barcelona	B1	331	5.4
	B2	2816	3.4

## Data Availability

The original data presented in the study are openly available in Zenodo at https://doi.org/10.5281/zenodo.17201713.

## Abbreviations

The following abbreviations are used in this manuscript:

ABP	Activation By Personalization
ACK	Acknowledgment
CR	Coding Rate
CSS	Chirp Spread Spectrum
DR	Data Rate
DtS-IoT	Direct-to-Satellite IoT
ED	End Device
FSK	Frequency Shift Keying
GMSK	Gaussian Minimum-Shift Keying
GW	Gateway
IoT	Internet of Things
LEO	Low Earth Orbit
LoRa	Long Range
LoRaWAN	Long Range Wide Area Network
LoS	Line-of-Sight
LR-FHSS	Long Range - Frequency Hopping Spread Spectrum
LPWAN	Low Power Wide Area Network
MAC	Media Access Control
MSE	Mean Squared Error
NLoS	Non-Line-of-Sight
NS	Network Server
NTN	Non-Terrestrial Network
OTAA	Over-the-Air Activation
PDR	Packet Delivery Ratio
PHY	Physical Layer
RSSI	Received Signal Strength Indicator
SF	Spreading Factor
SNR	Signal-to-Noise Ratio
ToA	Time-on-Air
